# Supplementary material for: CHD7 binds to insulators during neuronal differentiation
Source: bioRxiv. 2025 Mar 29:2025.03.28.646031. Preprint. [Version 1] doi: 10.1101/2025.03.28.646031 (PMC11974851; doi:10.1101/2025.03.28.646031)
Supplement: Supplement 1 [file NIHPP2025.03.28.646031v1-supplement-1.pdf]

bioRxiv preprint doi: https://doi.org/10.1101/2025.03.28.646031; this version posted March 29, 2025. The copyright holder for this preprint (which was not certified by peer review) is the author/funder, who has granted bioRxiv a license to display the preprint in perpetuity. It is made available under aCC-BY 4.0 International license.

Supplemental Figures

Fig. S1 Gene Ontology (GO) analysis on differentially expressed genes in proliferating iMOPs and iMOP-derived neurons

Circos plot displaying select GO term descriptions with differential expression levels of genes within the GO process in (A) proliferating iMOPs and (B) iMOP-derived neurons.

Fig. S2 Knockdown of *Chd7* in iMOP cells

Differentiating iMOP cells were transduced with lentivirus containing scrambled (scrb) shRNA or three different *Chd7* shRNAs (*Chd7* shRNA 1-3). Cells were selected in a blasticidin-containing medium, and the total RNA was harvested from the cultures. (A) qPCR for *Chd7* transcript levels was performed from transduced cells and normalized to scrb shRNA control (n=3). (B) Representative Western blot of CHD7 expression levels in *Chd7* shRNA1 transduced cells using ACTB as a loading control (n=3). (C) Quantification of protein levels from Western blot showing a decrease in CHD7 to 0.32-fold (n=3) in *Chd7* shRNA1 compared to scrb shRNA. (D) qPCR primer sequences. (E) shRNA containing oligos that were annealed, extended and inserted into cloning vectors.

Fig. S3 GO analysis on CTCF+CHD7+ enriched sites.

GO terms associated with progenitor-specific, common, and neuron-specific CTCF+CHD7+ enriched sites. GO terms were clustered into similar groups and displayed as a matrix using the simplifyEnrichment package. Word clouds on the right of the matrixes display the key words that are over-represented in each category.

Fig. S4 Targeting CTCF+ CHD7+ regions around *Mir9-2*

bioRxiv preprint doi: https://doi.org/10.1101/2025.03.28.646031; this version posted March 29, 2025. The copyright holder for this preprint (which was not certified by peer review) is the author/funder, who has granted bioRxiv a license to display the preprint in perpetuity. It is made available under aCC-BY 4.0 International license.

(A-B) Fluorescence of mCherry and eYFP from inducible dual fluorescent reporter plasmid containing no sgRNA (empty sgRNA) and individual sgRNAs in dCas9-KRAB-MeCP2 iMOP cells undergoing early-stage neuronal differentiation (n=3, independent experiments). sgRNA2 and sgRNA3 did not show significant changes in mCherry level across different eYFP fluorescence bins compared to empty sgRNA. (C-D) Fluorescence of mCherry and eYFP from inducible dual fluorescent reporter plasmid containing no sgRNA (empty sgRNA) and individual sgRNAs in dCas9-p300-core cells undergoing early-stage neuronal differentiation (n=4, independent experiments). sgRNA2 and sgRNA3 showed a statistically significant decrease in mCherry level compared to the control empty sgRNA in some eYFP fluorescence bins.

bioRxiv preprint doi: https://doi.org/10.1101/2025.03.28.646031; this version posted March 29, 2025. The copyright holder for this preprint (which was not certified by peer review) is the author/funder, who has granted bioRxiv a license to display the preprint in perpetuity. It is made available under aCC-BY 4.0 International license.

Fig. S1

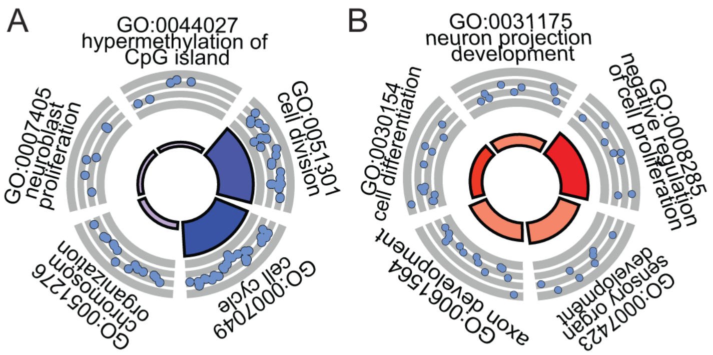

bioRxiv preprint doi: https://doi.org/10.1101/2025.03.28.646031; this version posted March 29, 2025. The copyright holder for this preprint (which was not certified by peer review) is the author/funder, who has granted bioRxiv a license to display the preprint in perpetuity. It is made available under aCC-BY 4.0 International license.

Fig. S2

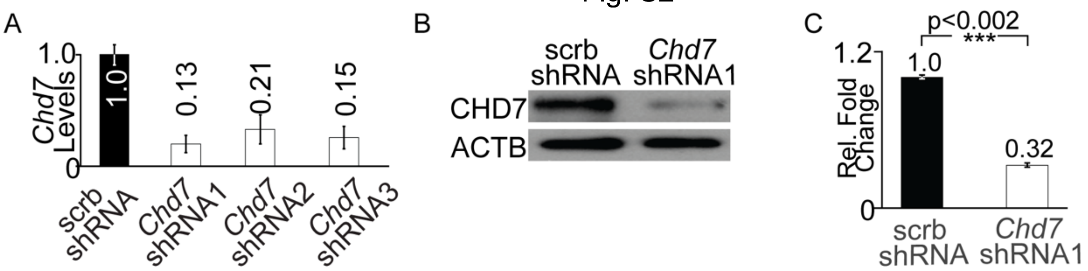

D

qPCR primers

Gene	Forward primer sequence (5'-3')	Reverse primer sequence (5'-3')
Chd7	AACCTGTCCTCCACTACAGC	TCACTAGCTGAGCGTTCTGT
Gapdh	AGGTCGGTGTGAACGGATTTG	TGTAGACCATGTAGTTGAGGTCA

E

Forward and reverse shRNA containing oligos were annealed and inserted into cloning vectors. Nucleotides labeled in red correspond to the shRNA sequences.

Cloning vector	shRNA	Forward sequence (5'-3')	Reverse sequence (5'-3')
pLKO.1-blast	Scrambled	CCGGCCTAAGGTTAAGTCGCCCTC<br>GCTCCTCGAGGAGCGAGGGCGAC<br>TTAACCTTAGGTTTTTG	AATTCAAAAACCTAAGGTTAAGTCGCCC<br>TCGCTCCTCGAGGAGCGAGGGCGACTT<br>AACCTTAGG
pLKO.1-blast	*Chd7* shRNA-1	CCGGGCCAGCCGTCGGACCATTC<br>CTCGAGGAATGGTCCGACGGCTG<br>GCTTTTTG	AATTCAAAAAGCCAGCCGTCGGACCATT<br>CCTCGAGGAATGGTCCGACGGCTGGC
pLKO.1-blast	*Chd7* shRNA-2	CCGGCTGTCCTGAGCTGCGTAATA<br>TCTCGAGATATTACGCAGCTCAGG<br>ACAGTTTTTG	AATTCAAAAACTGTCCTGAGCTGCGTAA<br>TATCTCGAGATATTACGCAGCTCAGGAC<br>AG
pLKO.1-blast	*Chd7* shRNA-3	CCGGGCCTATCAGCGCAGCTATAA<br>ACTCGAGTTTATAGCTGCGCTGAT<br>AGGCTTTTTG	AATTCAAAAAGCCTATCAGCGCAGCTAT<br>AAACTCGAGTTTATAGCTGCGCTGATAG<br>GC
pLKO.3G	Scrambled	AATTCCTAAGGTTAAGTCGCCCTC<br>GCTCGAGCGAGGGCGACTTAACCT<br>TAGGTTTTTTTA	AAAAAAACCTAAGGTTAAGTCGCCCTCG<br>CTCGAGCGAGGGCGACTTAACCTTAGG
pLKO.3G	*Chd7* shRNA-1	AATTGCCAGCCGTCGGACCATTCC<br>TCGAGGAATGGTCCGACGGCTGG<br>CTTTTTTTAT	AAAAAAAGCCAGCCGTCGGACCATTCC<br>TCGAGGAATGGTCCGACGGCTGGC

bioRxiv preprint doi: https://doi.org/10.1101/2025.03.28.646031; this version posted March 29, 2025. The copyright holder for this preprint (which was not certified by peer review) is the author/funder, who has granted bioRxiv a license to display the preprint in perpetuity. It is made available under aCC-BY 4.0 International license.

Fig. S3

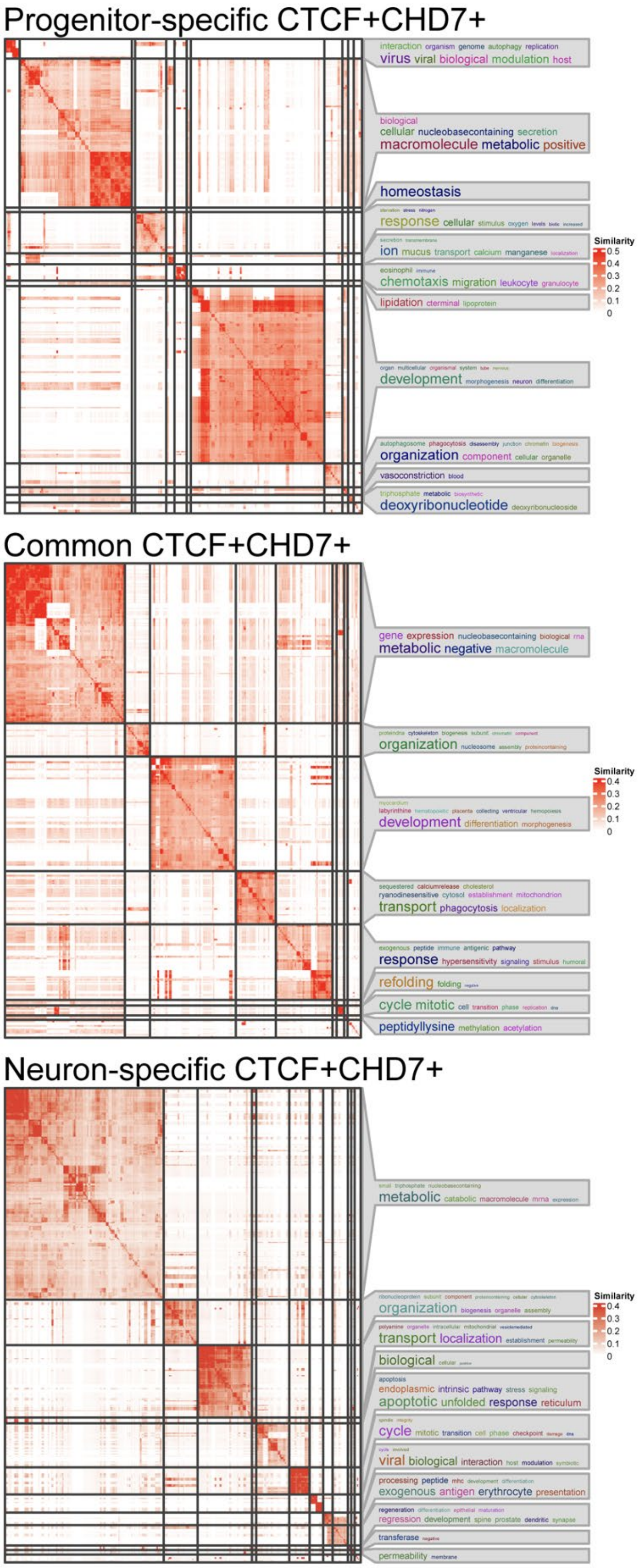

bioRxiv preprint doi: https://doi.org/10.1101/2025.03.28.646031; this version posted March 29, 2025. The copyright holder for this preprint (which was not certified by peer review) is the author/funder, who has granted bioRxiv a license to display the preprint in perpetuity. It is made available under aCC-BY 4.0 International license.

Fig. S4

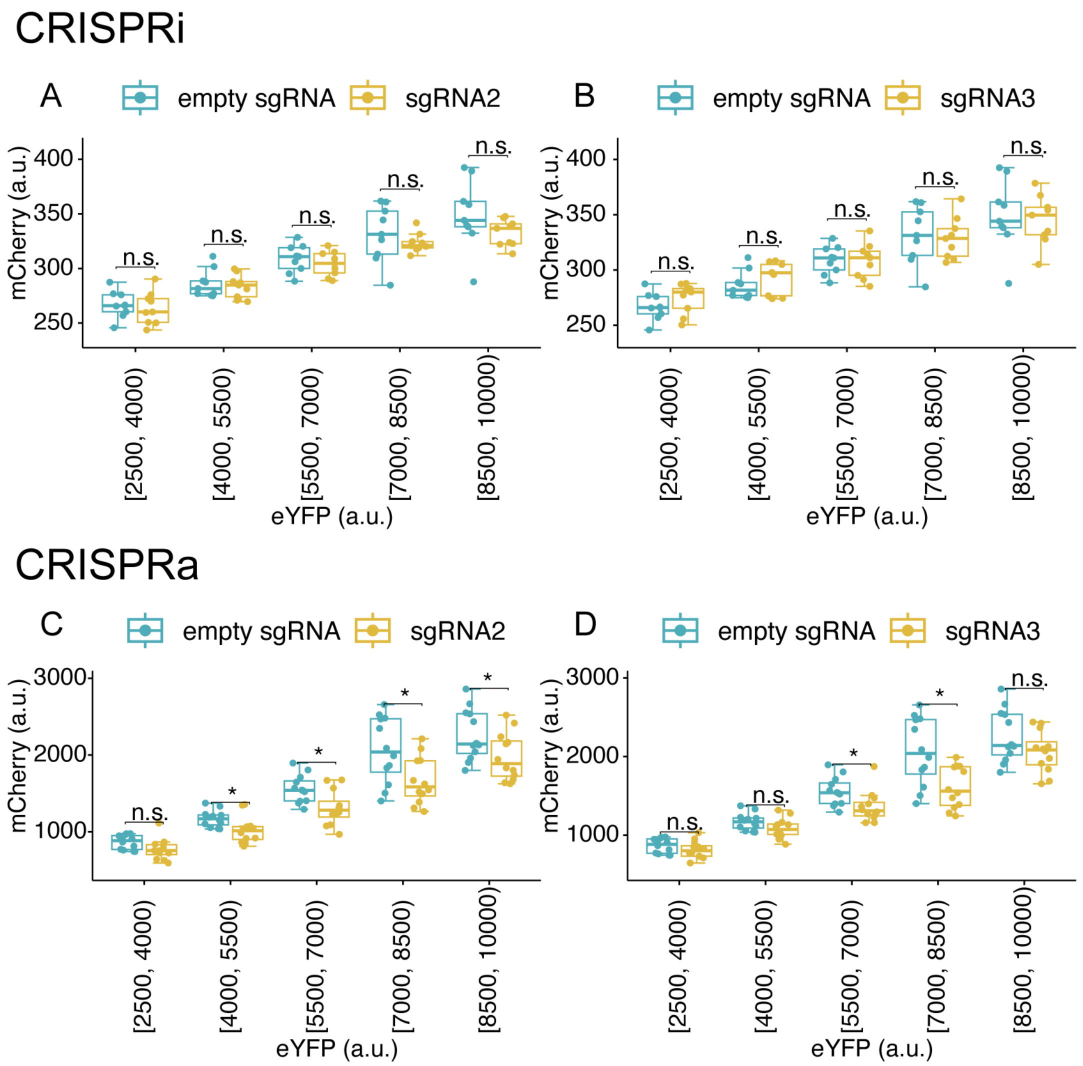

bioRxiv preprint doi: https://doi.org/10.1101/2025.03.28.646031; this version posted March 29, 2025. The copyright holder for this preprint (which was not certified by peer review) is the author/funder, who has granted bioRxiv a license to display the preprint in perpetuity. It is made available under aCC-BY 4.0 International license.

Table S1: sgRNAs used for targeting regions around *Mir9-2* using CRISPRi and CRISPRa

sgRNA name	sgRNA sequence (5' ->3')
sgRNA1	ACACCCGAGTCCATCAAGGC
sgRNA2	GGGGAGCAATTTATTCCTGG
sgRNA3	GGGCGCGCAAACAGGCCCGA
sgRNA4	GCTCCGATTCCCACGCTCGG
sgRNA5	CCAGAGATCTAAAAATAGCC

bioRxiv preprint doi: https://doi.org/10.1101/2025.03.28.646031; this version posted March 29, 2025. The copyright holder for this preprint (which was not certified by peer review) is the author/funder, who has granted bioRxiv a license to display the preprint in perpetuity. It is made available under aCC-BY 4.0 International license.

Table S2

Reagent	Source	Identifier	Concentration /Dilution	Application
Mouse anti-TUBB3	BioLegend	Cat# 801202, RRID:AB_2313773	1:1000	Immunostaining
Rabbit anti-TUBB3	BioLegend	Cat# 845501, RRID:AB_2566588	1:1000	Immunostaining
Mouse anti-CDKN1B	Thermofisher/Lab Vision	Cat# MS-256-P RRID:AB_61871	1:500	Immunostaining
Rabbit anti-CHD7	Cell Signaling Technology	Cat# 6505, RRID:AB 11220431	1:300	Immunostaining
Alexa Fluor 568 Phalloidin	Thermo Fisher Scientific	Cat# A12380	1:40	Immunostaining
Goat anti-rabbit Alexa Fluor 488	Thermo Fisher Scientific	Cat# A-11034, RRID:AB_2576217	1:3000	Immunostaining
Goat anti-mouse Alexa Fluor 568	Thermo Fisher Scientific	Cat# A-11031, RRID:AB_144696	1:3000	Immunostaining
Goat anti-rabbit Alexa Fluor 647	Thermo Fisher Scientific	Cat# A-21244, RRID:AB_2535812	1:3000	Immunostaining
Rabbit anti-EP300	Santa Cruz Biotechnology	Cat# sc-585, RRID:AB_2231120	1:50	CUT&Tag
Rabbit anti-H3K4me3	Active Motif	Cat# 39159, RRID: AB_2615077	1:50	CUT&Tag
Rabbit anti-CHD7	Cell Signaling Technology	Cat# 6505, RRID:AB_11220431	1:50	CUT&Tag
Rabbit anti-CTCF	Active Motif	Cat# 61311, RRID:AB_2614975	1:50	CUT&Tag
